# Primary squamous cell carcinoma of the pancreas: an update on a rare neoplasm from the SEER database

**DOI:** 10.3389/fonc.2023.1272740

**Published:** 2023-12-07

**Authors:** Jacob A. Ford, Arjun Bhatt, Rachel C. Kim, Michael Larkins, Aidan M. Burke

**Affiliations:** ^1^ Brody School of Medicine, East Carolina University, Greenville, NC, United States; ^2^ Department of Surgery, Indiana University School of Medicine, Indianapolis, IN, United States; ^3^ Department of Radiation Oncology, East Carolina University (ECU) Health, Greenville, NC, United States

**Keywords:** squamous cell carcinoma of the pancreas, oligometastatic condition, histology, surgery, SEER

## Abstract

**Introduction:**

Pancreatic squamous cell carcinoma is a rare type of pancreatic cancer of ductal origin, composing an estimated 0.5 - 5% of pancreatic ductal malignancies. As a result, epidemiology, treatment options, and associated outcomes are poorly understood and understudied. Our aim was two-fold: to evaluate demographic trends and analyze overall survival (OS) associated with different treatment modalities for this rare malignancy.

**Methods:**

Patients with pancreatic squamous cell carcinoma diagnosed between 1992 and 2019 were eligible and reviewed utilizing the Surveillance, Epidemiology, and End Results Registry (SEER) database. Data was analyzed using SPSS and python packages lifelines and pandas. Variables of interest included stage at diagnosis as well as the receipt of surgery, radiotherapy, and/or chemotherapy. Five-year OS curves were analyzed using Kaplan-Meier probability stratified by treatment modality.

**Results:**

Of 342 cases of pancreatic squamous cell carcinoma, 170 (49.7%) were females and 172 (50.3%) were males. 72 (21.1%) of patients received radiotherapy, 123 (35.9%) patients received chemotherapy, and 47 (13.7%) received surgery. Patients who were diagnosed under the age of 50 had prolonged survival time compared to those diagnosed over the age of 50 (12 vs 8 months, respectively, p < 0.001). This trend was evident despite the lack of a significant association between age at diagnosis and presence of metastases (p = 0.524). The median OS was 3 months for the entire cohort and there was a significant difference in median survival time noted across treatment modalities: OS was prolonged in those receiving surgery compared to those receiving chemotherapy or radiotherapy alone (30 vs 2 months, respectively, (p<0.001)). Receipt of radiotherapy was not associated with a significant difference in OS compared to those who did not receive radiotherapy.

**Conclusion:**

Pancreatic squamous cell carcinoma is a rare subtype of pancreatic cancer and typically portends a poor prognosis. As demonstrated by our study, surgery offers prolonged overall survival compared to other treatment modalities. Age at diagnosis and presence of metastatic disease are also important prognostic factors likely related to patients‘ ability to tolerate surgery or physician willingness to offer surgery. Given the importance of surgery on outcomes, it may be reasonable to offer it in the oligometastatic setting in patients who are otherwise a good candidate. Future research on larger cohorts is warranted to investigate the role that modality selection plays in overall survival rates in this understudied malignancy.

## 1 Introduction

Primary cancers of the pancreas are recognized widely for their poor outcomes (1). This can be attributed, in part, to the lack of effective screening methods and are thus often unresectable at diagnosis, which makes definitive therapy difficult if not impossible as surgical excision continues to be required for curative treatment (2). Furthermore, the anatomic location of pancreatic cancers in relation to vital mesenteric vascular structures make excision technically demanding and at high risk for complications (1–3).

Pancreatic cancer discussions often assume a histological diagnosis of primary pancreatic ductal adenocarcinoma (PDAC), however, there is an important and often underappreciated understanding of other histologic types of pancreatic cancer, particularly primary squamous cell carcinoma of the pancreas (PSCC). Primary squamous cell carcinoma of the pancreas makes up between 0.5 - 5 percent of primary pancreatic tumors, and recent evidence may suggest this incidence is increasing with time (4–6). In addition, there may be clinical importance to the evaluation of subtypes within PSCC based on histology including papillary, spindle cell, and clear cell morphologies (7). **Table 1** provides an overview of suggested subtypes of primary squamous cell carcinoma of the pancreas with reference material including photomicrographic demonstration of these subtypes.

**Table 1:** Reference material to histologic subtypes of PSCC.

Subtype	Reference Example from Literature
PSCC NOS (9)	https://pubmed.ncbi.nlm.nih.gov/30313026/
Basaloid (10)	https://pubmed.ncbi.nlm.nih.gov/22973995/
Keratinizing (11)	https://pubmed.ncbi.nlm.nih.gov/28184374/
Clear Cell (12)	https://pubmed.ncbi.nlm.nih.gov/34619878/
Spindle Cell/Sarcomatoid (13)	https://pubmed.ncbi.nlm.nih.gov/30363708/
Papillary (14)	https://pubmed.ncbi.nlm.nih.gov/30388929/
Adenoid (15)	https://pubmed.ncbi.nlm.nih.gov/36924068/

In comparison to PDAC, PSCC is associated with poor differentiation, more aggressive behavior, and worse overall outcomes (1, 8). Patients diagnosed with PSCC are more likely to be male, black, and older in age relative to the general US population, which is consistent with PDAC. However, acquired risk factors for PDAC, such as smoking and chronic pancreatitis, do not seem to be associated with PSCC (16–18).

Treatment for PSCC closely resembles treatment of PDAC with surgical resection as the gold standard for curative treatment in non-metastatic and resectable cases (1, 4). The role of neoadjuvant chemotherapy is not well understood; this may be a reflection of a low sample size as most cases of this rare disease are treated with primary urgent and early surgical resection with curative intent rather than neoadjuvant therapeutic combinations (5, 19). The rapid growth of these neoplasms likely precludes neoadjuvant treatment at our current level of understanding.

Additionally, PSCC is often metastatic at the time of diagnosis. This leaves most patients with palliative, rather than curative, treatment options (4, 18). Finding more avenues for curative treatment is a major goal in research for this aggressive cancer type. More information is needed to determine if curative surgical resection may be a possible treatment plan in the setting of oligometastatic disease, particularly in patients with metastasis only to the liver as in the treatment of colorectal carcinoma, as such analyses of oligometastatic disease of PSCC have historically been difficult due to the rarity of the disease and low sample size (1, 18, 20).

Previous SEER studies have compared PSCC to PDAC, and found negative prognostic implications of advanced disease Stage, and comparatively increased grade among PSCC (1). Other studies have focused on the precise location of PSCC within the pancreas, finding body and tail lesions associated with decreased survival, and overlapping lesions associated with even larger decreases in survival (1). (2) It is theorized that overlapping lesions may correspond to particular histological subtypes of PSCC, but an analysis of PSCC subtypes was out of the scope of their investigation.

The aim of this study is to provide an update to the body of knowledge on primary squamous cell carcinoma of the pancreas by utilizing the SEER database to stratify survival outcomes by histologic subtype, as well as to update previous SEER studies with the new 2018-2019 SEER data. This update triples the present study’s sample size, allowing for further analysis of the distribution of metastatic disease, grade, tumor size, and demographic data. We also aim to provide a novel subgroup analysis of patients with metastatic disease and their treatment outcomes utilizing the updated SEER data.

## 2 Methods

Patients with primary pancreatic squamous cell carcinoma diagnosed between 1992 and 2019 were eligible and reviewed utilizing the Surveillance, Epidemiology, and End Results Registry (SEER) database. The primary outcome was differences in overall survival based on treatment types. Data was analyzed using SPSS and Python packages *lifelines* and *pandas*. Variables of interest included stage at diagnosis as well as the receipt of surgery, radiotherapy, and/or chemotherapy. Patient characteristics included age at diagnosis, sex, and race. Tumor characteristics included histologic subtype, size, grade, presence of metastatic disease, and site of metastatic disease. Kaplan Meier survival analysis was used to compare overall survival across multiple independent variables. Statistically significant differences in survival were based on univariate analysis and a multivariate Cox proportional hazards analysis, done through Breslow’s method as implemented in Python package lifelines. Multivariate analysis investigated variables such as metastatic condition (recoded as a binary), grade, race, patient age, the presence of and time relation of chemotherapy and radiation relative to surgery, and histologic type; for both univariate and multivariate analysis differences were reported as p values at a significance value of <0.05. In addition to the primary outcome, survival outcomes of patients who specifically had metastatic disease present at the time of diagnosis were also examined.

## 3 Results

### 3.1 Clinical trends

#### 3.1.1 Patient demographics

Of 342 cases of pancreatic squamous cell carcinoma, 170 (49.7%) were females and 172 (50.3%) were males. As expected, PSCC patients tended to be older with the largest cohort of patients in the 70-79 age group (31.0%). Fifteen percent of patients were Black, 73.2% White, and 11.7% were of other racial groups. Patient characteristics are shown below in **Table 2**.

**Table 2:** Patient characteristics.

PSCC Patients (n=342)		N	%
Age	<30	7	1.6%
	30-39	4	0.9%
	40-49	29	6.9%
	50-59	68	16.2%
	60-69	94	22.4%
	70-79	130	31.0%
	80+	87	20.7%
Sex	Female	170	49.7%
	Male	172	50.3
Race	Black	63	15.0%
	White	307	73.2%
	Other Race	49	11.7%

#### 3.1.2 Tumor characteristics

**Figure 1:**
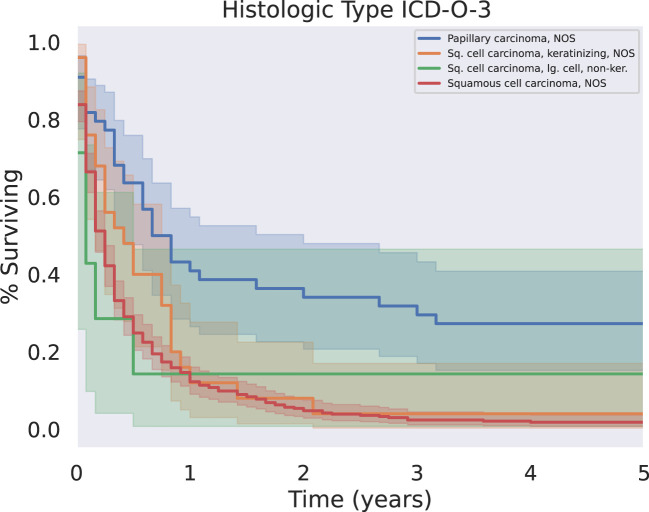
KM Survival across the four most common histologic subtypes. Papillary carcinoma subtypes were associated with significantly longer five year survival than other squamous cell carcinoma subtypes (p < 0.05).

Our analysis investigated tumor grade, size, metastatic disease, and histologic subtype as aspects of the tumor itself that may impact outcomes. These results are summarized in **Table 3** and **Figure 1**.

**Table 3:** Tumor characteristics.

Tumor Characteristics		N	%
Grade	Well Differentiated	10	2.4%
	Moderately Differentiated	32	7.6%
	Poorly Differentiated	115	27.5%
	Unknown	225	53.7%
	Not Reported	37	8.8%
Tumor Size	<4 cm	144	34.4%
	>4 cm	227	54.2%
	Unknown	48	11.5%
Metastatic Disease	Local Only	117	27.9%
	Metastatic Disease	133	31.7%
	Unknown	169	40.3%

##### 3.1.2.1 Grade

In our analysis of the SEER database, 2.4% of reported PSCC cases were considered well differentiated, while 27.5% of cases were poorly differentiated. 53.7% of cases had an unknown or not recorded tumor grade (53.7%).

##### 3.1.2.2 Metastatic condition

A significant proportion of the study population had metastatic disease at the time of diagnosis (31.7%). Again, a significant segment of the study population was reported as unknown metastatic condition at the time of diagnosis (40.3%).

##### 3.1.2.3 Histologic subtype

Approximately 80% of cases were described as “Squamous cell carcinoma, NOS”. Some cancers were further differentiated into papillary carcinoma (10.5%), keratinizing PSCC (6.0%), and large-cell non-keratinizing PSCC (1.7%). There were also even rarer subtypes discovered, such as spindle cell PSCC (0.5%) and basaloid PSCC (0.5%).

#### 3.1.3 Treatment

**Figure 2:**
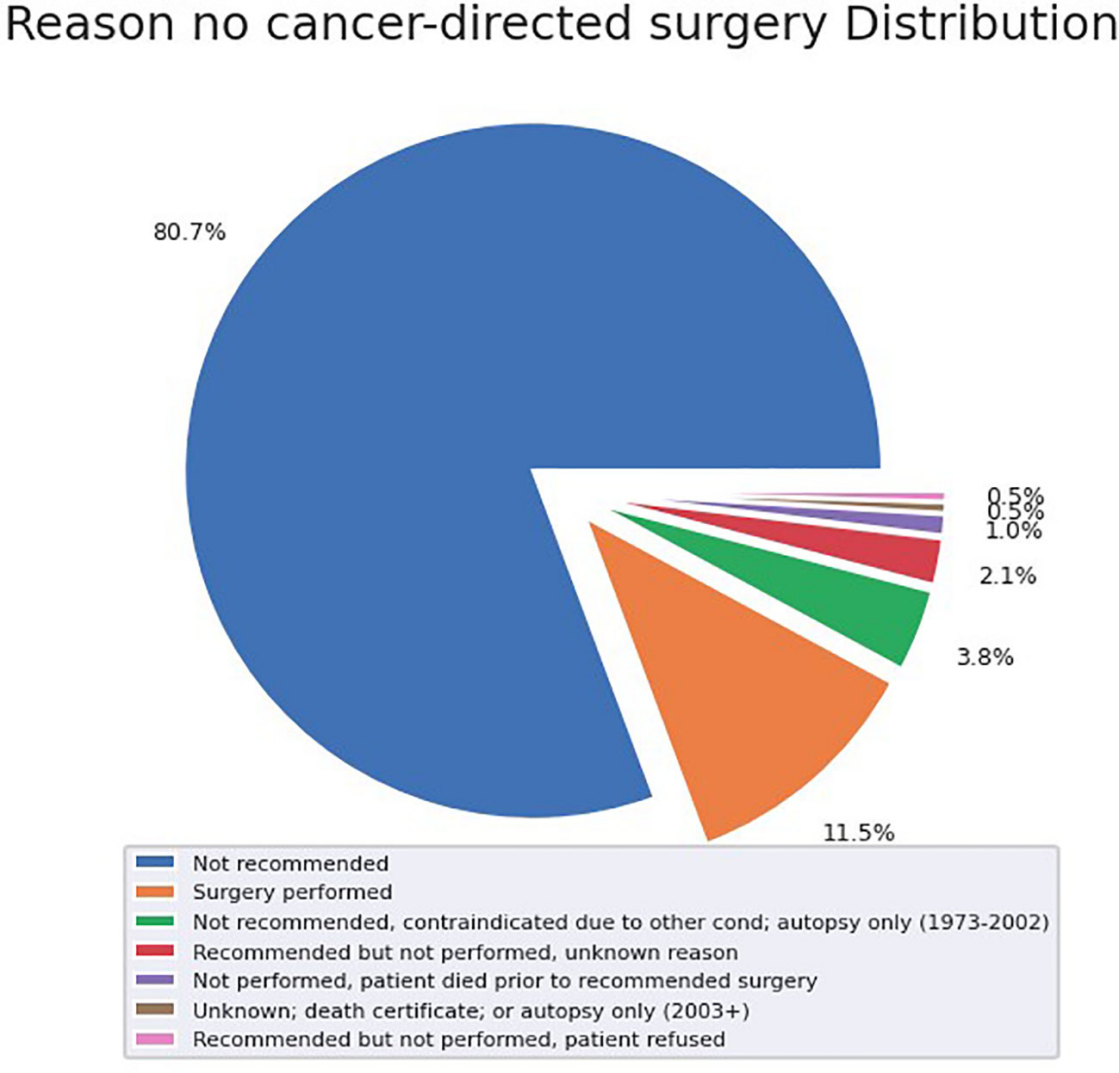
Surgical treatment & reason for no cancer-directed surgery.

72 (21.1%) patients received radiotherapy, 123 (35.9%) patients received chemotherapy, and 47 (13.7%) received surgery. Of the patients who did not undergo surgical resection, 80.7% did not receive surgery because it was not recommended based on their tumor characteristics and surgical location (**Figure 2**). When specifically examining patients with metastatic disease, this proportion of patients who were not recommended surgery increased to 94.8% of tumors.

### 3.2 Survival/outcomes analysis

#### 3.2.1 Demographics

**Figure 3:**
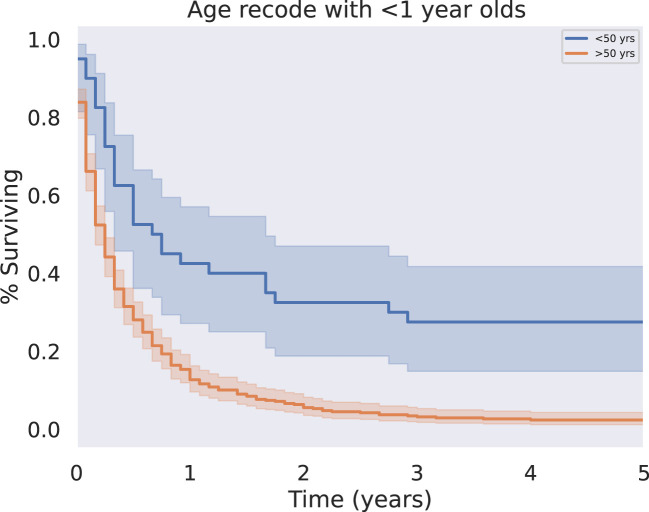
KM Survival Age, Above or below 50 years. Patient age was analyzed as a binary variable, separating patients less than or equal to fifty from those greater than or equal to fifty years old. Younger patients had significantly improved five year overall survival outcomes relative to old (p < 0.05).

Patients who were diagnosed under the age of 50 had prolonged survival time compared to those diagnosed over the age of 50 (median survival 8.5 vs 3 months, respectively, p < 0.001) (**Figure 3**). This trend was evident despite the lack of a significant association between age at diagnosis and presence of metastases (p = 0.524). Other demographic variables such as race were not significant predictors of survival.

#### 3.2.2 Tumor characteristics

##### 3.2.2.1 Grade

**Figure 4:**
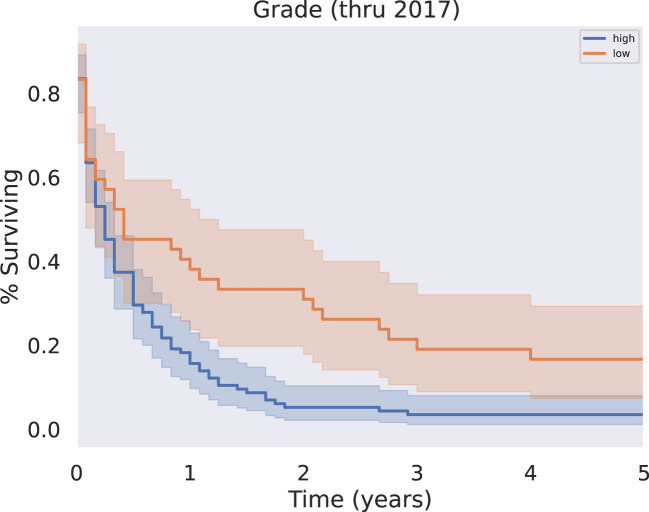
KM Survival, High vs Low Grade. Grade, originally coded as I through IV, was analyzed as either low grade (I-II) or high grade (III-IV). Survival analysis revealed a significant survival benefit associated with a diagnosis of low grade rather than high grade disease (p < 0.05).

Patients with tumors reported as low grade (well differentiated or moderately differentiated) at the time of diagnosis had improved overall survival compared with patients with a “high grade” tumor classification (poorly differentiated or undifferentiated) at the time of diagnosis (p<0.05) (**Figure 4**). Patients with higher grade disease were also more likely to have metastases, as in 25.1% of these patients, compared to 9.1% of patients with lower grade disease (p = 0.0477). This further suggests patients with higher grade disease may be less likely to be surgical candidates.

##### 3.2.2.2 Histology

Patients with tumors further subclassified into papillary carcinoma had significantly prolonged survival relative to all other histologies (**Figure 1**, p < 0.05). There was only one case of the clear cell subtype of squamous cell carcinoma; this patient did not survive past 1 month from the time of their diagnosis.

#### 3.2.3 Treatment modalities

The benefit of surgery, chemotherapy, and radiation were analyzed overall and specifically in the setting of metastatic disease.

##### 3.2.3.1 Surgery and chemoradiation

**Figure 5:**
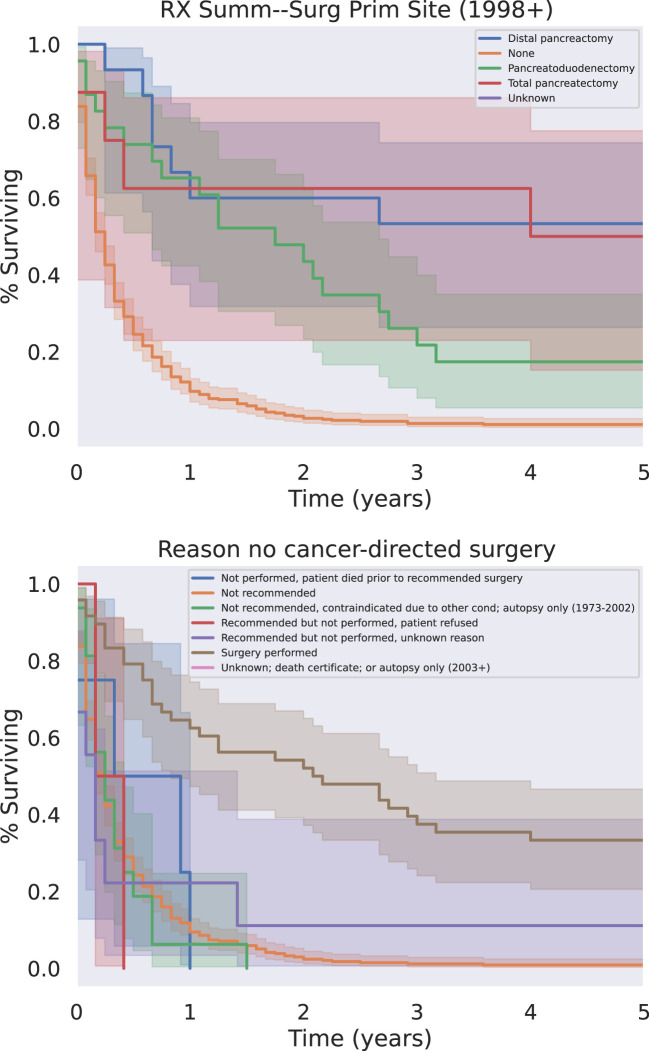
Surgery Type (Top) And/Or Reason Why Not (Bottom). All types of surgery demonstrated a significant survival benefit relative to the absence of surgery regardless of the reason the patient did not receive surgery (p < 0.05).

**Figure 6:**
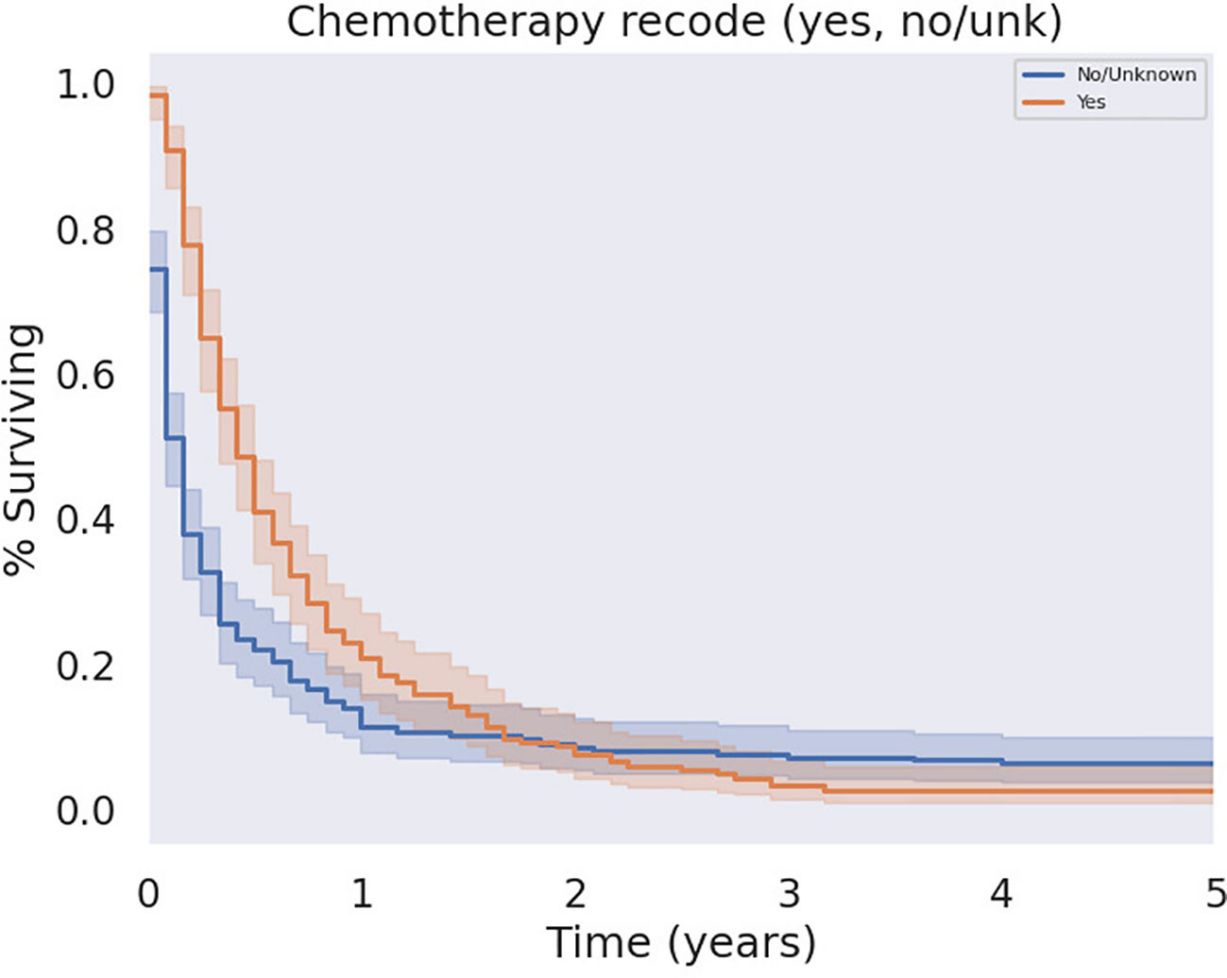
KM Curve of Systemic Chemotherapy Received. Univariate analysis of all patients who received chemotherapy versus those who did not failed to demonstrate a statistically significant survival benefit associated with chemotherapy (p = 0.062).

**Figure 7:**
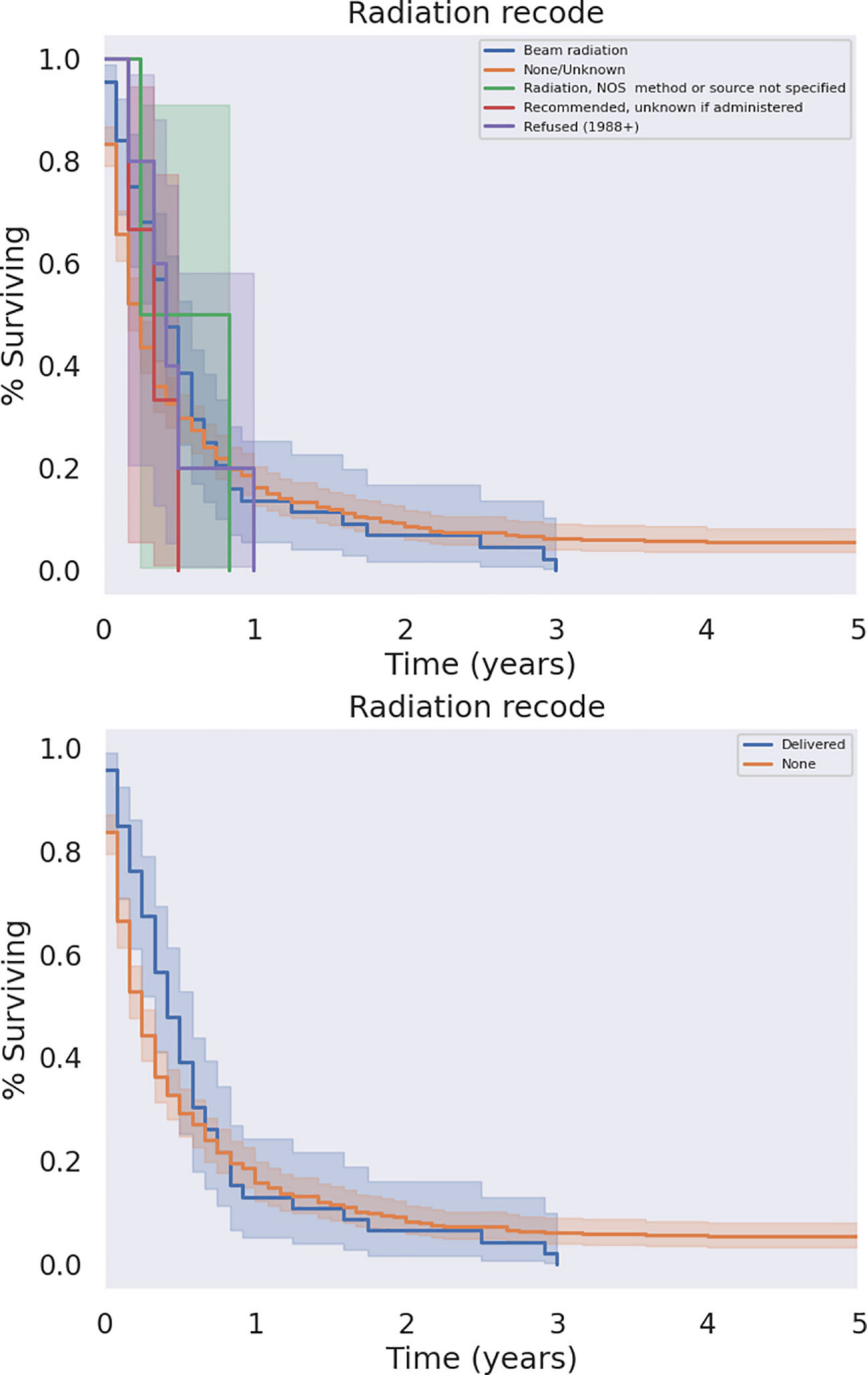
KM Curve of Radiation Therapy Received. Univariate analysis of all patients who received radiation versus those who did not failed to demonstrate a statistically significant survival benefit associated with radiation (p = 0.079).

The modalities of treatment for PSCC included surgical resection, chemotherapy, and radiotherapy, as well as combinations of the three. The median OS was 3 months for the entire cohort and there was a significant difference in median survival time noted across treatment modalities. There was a clear benefit to overall survival in patients who received surgical intervention (**Figure 5**, p<0.05). The type of surgery, including local excision, partial pancreatectomy, pancreaticoduodenectomy with or without gastrectomy, or total pancreatectomy, did not have a significant effect on outcomes. Patients who received chemotherapy demonstrated no significant improvement in overall median survival (p = 0.062) compared to those who did not (**Figure 6**). Radiation treatment also did not have a significant effect on patient survival (**Figure 7**). When comparing treatment modalities, median OS was prolonged in those receiving surgery (median OS 30 months) compared to those receiving chemotherapy (median OS 2 months) or radiotherapy (median OS 2 months) alone (p<0.001)).

##### 3.2.3.2 Adjuvant vs neoadjuvant treatment

**Figure 8:**
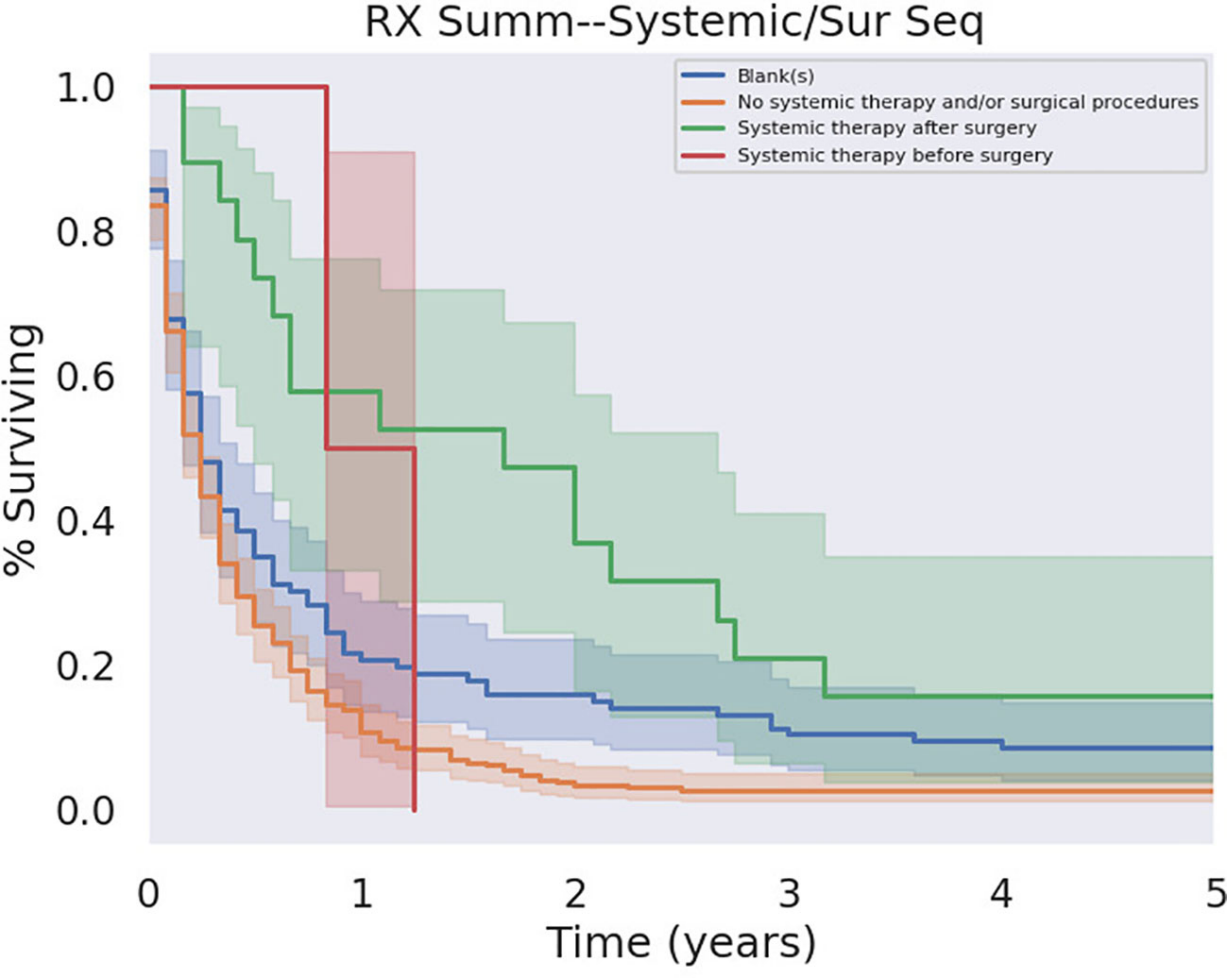
Adjuvant versus Neoadjuvant Chemotherapy. Adjuvant chemotherapy demonstrated a significant survival benefit over its absence ce (p < 0.05).

Comparing the timing of chemotherapy treatment in combination with surgical resection of PSCC, there was a benefit in OS for patients receiving adjuvant chemotherapy relative to patients who did not receive chemotherapy at all (p<0.05) as well as relative to patients who received neoadjuvant chemotherapy (p < 0.05). In contrast, neoadjuvant chemotherapy did not significantly affect outcomes relative to no chemotherapy at all (p=0.12). Survival in relation to adjuvant and neoadjuvant therapy is illustrated in **Figure 8**.

#### 3.2.4 Metastatic disease

**Figure 9:**
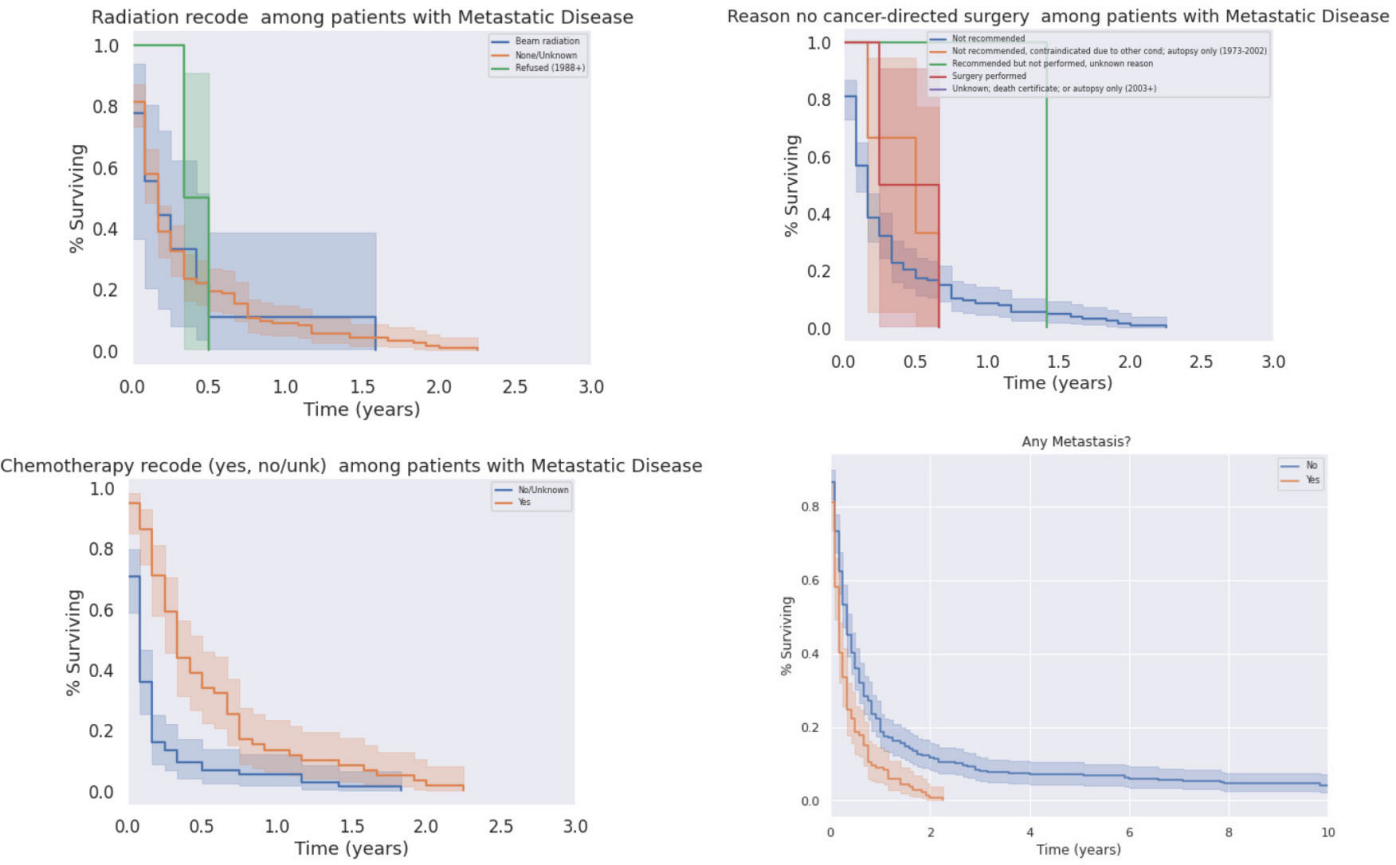
Impacts of Radiation (Top Left), Chemotherapy (Bottom Left), and Surgery (Top Right) on Survival in the Metastatic Condition. Chemotherapy significantly prolonged survival in patients in metastatic disease (p < 0.05), who otherwise had significantly decreased survival relative to patients without metastases (p < 0.05).

We analyzed survival outcomes based on treatment modality within metastatic PSCC patients. Only half (49.3%) of patients with metastatic disease at diagnosis underwent treatment of any kind, compared to patients without metastases (59.1%). Notably, surgical intervention did not yield a significant increase in overall survival amongst patients with metastatic disease. Similarly, radiation treatment did not significantly affect outcomes. In contrast, patients with metastatic disease who were treated with chemotherapy did experience survival benefit (**Figure 9**, p<0.05). As expected, patients with metastatic disease at the time of diagnosis had worse outcomes than patients without metastatic disease; there was a poor prognosis with minimally variable differences in median survival between liver only metastases and distant metastases (both with median of 2 months, decrease in mean survival by 0.4 months). A survey of metastatic disease sites, as well as treatment modalities used for patients with metastatic disease, is presented below in **Table 4**; Kaplan-Meier curves for treatment in the metastatic condition are shown in **Figure 9**.

**Table 4:** Characteristics of metastatic disease.

							Race			Treatment			Survival
Location of Metastasis	Total Number (n)	40-50	50-60	60-70	70-80	80+	White %	Black %	Other Race %	%Surgery	%Chemo	%Radiation	Survival in months (median, mean)
Liver Alone	96	9.50%	21.10%	18.90%	38.90%	12.60%	73.70%	16.80%	10.50%	1.5%	44.88%	7.1%	2.0, 4.2
Distant Metastases	33	7.12%	19.87%	19.87%	39.34%	14.17%	63.63%	15.15%	21.2%	3%	39.50%	18.20%	2.0, 3.8
Present but unspecified	5	0.00%	0.00%	0.00%	60.00%	40.00%	100.00%	0.00%	0.00%	0.00%	0.00%	0.00%	1.0, 1.2

## 4 Discussion

Pancreatic squamous cell carcinoma is a rare subtype of pancreatic cancer and typically portends a poor prognosis. This rare cancer is often poorly differentiated, fast-growing, and even metastatic at the time of diagnosis (1, 4, 6). For pancreatic adenocarcinoma it is estimated that the mean time from Stage I to Stage IV disease is 1.3 years, which emphasizes the importance of decisive and prompt action in the often more aggressive SCC form (4).

In 2016, Makarova-Rusher et al. utilized the SEER database to examine the cases of 214 patients with primary SCC of the pancreas comparing overall survival across treatment types to primary pancreatic adenocarcinoma. They reported an overall survival of 14% at one year and 7% at two years. Patients who received surgical resection had an overall survival of 45% at one year and 35% at two years (4). Our analysis includes the updated SEER data which demonstrates the same trends and additionally considers the role of metastatic disease at the time of diagnosis as well as histologic subtype. In 2017, Ntanasis-Stathopoulos et al. performed a systematic review on the topic which found a trend of improving survival rates over time potentially associated with improved surgical techniques and more cancers being deemed resectable (1). Our study builds on this work to include additional characterization of PSCC of the pancreas to include histologic subtype and a subgroup analysis of patients with metastatic disease.

Histologic subtypes of pancreatic squamous cell carcinoma, including basaloid, papillary, keratinizing, spindle cell, adenoid, and clear cell squamous cell carcinoma have been poorly studied.14 Primary squamous cell carcinoma of the pancreas is exceedingly rare on its own which limits the characterization of these histologic subtypes to individual case studies and the interpretation of individual pathologists.

The sample of patients with these reported subtypes has historically been too low for statistical power, however, the present examination suggests the papillary subtype may have marginally better outcomes with a median survival of 9 months, approximately 6 months longer than the median survival for the cohort of pancreatic squamous cell carcinoma patients as a whole. While this still represents aggressive disease, and future research is needed to evaluate whether different histological subtypes are best treated with different modality permutations, there may be prognostic utility in further histologic subtyping.

Differentiation by histologic subtype is also complicated by the presence of mixed adenosquamous carcinoma of the pancreas which is defined as a ductal neoplasm with at least 30% of a squamous component (21). In comparison, primary squamous cell carcinoma of the pancreas is even more rare and is made up of purely squamous cells (22). While the SEER database does differentiate these subtypes of epithelial pancreatic cancer, the interpretation of a given neoplasm as squamous or adenosquamous may vary based on the reading pathologist (23). As only the final histological diagnosis of the pathologist is coded, and diagnostic detail and/or histologic features to support that diagnosis are not included, this study is necessarily limited by the diagnostic definitions used by the reading pathologist at the time of data reporting.

Based on our overall results, and consensus among available literature, surgery remains the most effective treatment modality for patients with resectable disease. Surgery offers prolonged overall survival and is the only potential curative treatment modality. We attempted to examine if the type/extent of surgery had an effect on survival or cause of death. With the limited information available, there was no clear difference based on the type of surgical intervention and this decision must be based on individual tumor location and characteristics. The role of neoadjuvant therapy in the treatment of pSCC remains poorly studied, likely due to the rarity of this disease, but our results suggest adjuvant therapy may yield better outcomes than neoadjuvant therapy. This may emphasize the central role of surgical treatment and suggests surgical treatment should generally not be delayed to prioritize other therapeutic modalities. Further research may investigate whether adjuvant chemotherapy yields superior outcomes over patients receiving surgery alone.

Other prognostic factors affecting overall survival include age at diagnosis and the presence of metastatic disease. These variables may interact with survival through the receipt of surgery: the present study found both that patients with metastatic disease are typically not offered surgery, and that older patients were more likely to not be offered surgery. These two populations correspondingly had the worst survival. The two principal reasons cited for elderly patients not being offered surgery were poor surgical candidacy and other medical conditions.

Among patients with metastatic disease at the time of diagnosis, we found that overall survival outcomes were significantly worse (p < 0.05). A large majority (71.6%) of cases were only metastatic to the liver at the time of diagnosis, which identifies this population as a potential target for adaptation and improvement of treatment modalities. This hepatic metastasis population, however, still had poor survival, similar to those with extrahepatic metastases. Among patients with metastatic disease, surgery did not significantly improve survival compared to non-surgical intervention, while there was a significant survival benefit in patients with metastases who received chemotherapy. It is unclear from this dataset, however, if the intent of surgery in metastatic patients was palliative versus cytoreductive. Given the importance of surgery on overall survival in PSCC it may be reasonable to offer surgical intervention in the oligometastatic setting in patients who are otherwise a good candidate. Future research on larger cohorts and randomized controlled trials are necessary to investigate the role modality selection plays in overall survival rates in this understudied malignancy.

Limitations of this study include those inherent to the SEER database, including incomplete data in many fields and lack of granularity. Specifically, it is impossible to distinguish with the SEER database whether patients did not undergo surgery due to unresectable tumor burden or because of patient fitness or presence of metastatic disease. The SEER database also does not allow for stratification or controlling by comorbid conditions. Confounding variables to our data analysis include the previously described data not collected by the SEER including individual factors such as surgical candidacy based on comorbid conditions. Finally, the study population covers a limited region restricted to a portion of the US participating in the database which limits the ability to generalize to larger populations. The ability to affect clinical decision making is limited by the omission of specific treatment methods beyond treatment modality as type of chemotherapy or radiation treatment are not included.

Nonetheless, the present SEER database study is to the authors knowledge the largest study of squamous cell carcinoma of the pancreas to date, and the first to specifically examine survival differentials across therapeutic modality and in the setting of liver-only versus distant metastatic disease. As discussed, most cases of pancreatic squamous cell carcinoma are metastatic at the time of diagnosis; these cases are the most likely to have high grade disease. By including a focus on this population, we aim to expand clinical understanding of current treatment modalities for these patients and their resulting overall survival. Furthermore, our analysis suggests that histologic subtype analysis of squamous cell carcinomas of the pancreas may yield improved prognostic information, demonstrating concretely that patients with papillary subtypes tend to survive longer. Finally, our study reaffirms the value of surgical therapy, including both local excision and total pancreatectomy, as the most effective treatment of this rare disease.

## Data availability statement

The original contributions presented in the study are included in the article/**Supplementary Material**. Further inquiries can be directed to the corresponding authors.

## Ethics statement

Ethical approval was not required for the study involving humans in accordance with the local legislation and institutional requirements. Written informed consent to participate in this study was not required from the participants or the participants’ legal guardians/next of kin in accordance with the national legislation and the institutional requirements.

## Author contributions

JF: Data curation, Formal Analysis, Investigation, Methodology, Writing – original draft, Writing – review and editing. ArB: Conceptualization, Data curation, Formal Analysis, Investigation, Methodology, Project administration, Software, Supervision, Validation, Visualization, Writing – review and editing. RK: Writing – review and editing. ML: Data curation, Investigation, Methodology, Supervision, Writing – review and editing. AiB: Writing – review and editing.

## Supplementary material

Supplementary Table 1 Multivariate analysis results.
