# Supplementary material for: Primary squamous cell carcinoma of the pancreas: an update on a rare neoplasm from the SEER database
Source: Front Oncol. 2023 Dec 7;13:1272740. doi: 10.3389/fonc.2023.1272740 (PMC10733453; doi:10.3389/fonc.2023.1272740)
Supplement: Supplementary Table 1 — Multivariate analysis results. [file Table_1.docx]

Supplementary Table 1: Multivariate Analysis

| **Multivariate Analysis** | **exp(coef)** | **se(coef)** | **coef lower 95%** | **coef upper 95%** | **exp(coef) lower 95%** | **exp(coef) upper 95%** | **cmp to** | **z** | **p** | **-log2(p)** |
| --- | --- | --- | --- | --- | --- | --- | --- | --- | --- | --- |
| Any Metastasis? | 6.62 | 751.46 | 1.17 | 4.33 | 8.91 | 76.1 | 7420.73 | 5.67 | <0.005 | 26.04 |
| Grade | 0.72 | 2.06 | 0.92 | -1.09 | 2.53 | 0.34 | 12.54 | 0.78 | 0.43 | 1.21 |
| Age | 3.49 | 32.73 | 1.27 | 1.01 | 5.97 | 2.73 | 392.02 | 2.75 | 0.01 | 7.41 |
| Race | -0.35 | 0.7 | 1.02 | -2.36 | 1.65 | 0.09 | 5.2 | -0.35 | 0.73 | 0.46 |
| Surgery/Radiation Sequence | -0.98 | 0.38 | 1.46 | -3.84 | 1.88 | 0.02 | 6.56 | -0.67 | 0.5 | 0.99 |
| Surgery/Chemotherapy Sequence | -2.94 | 0.05 | 1.08 | -5.06 | -0.82 | 0.01 | 0.44 | -2.72 | 0.01 | 7.26 |
| Histologic Subtype | 4.68 | 107.44 | 1.25 | 2.23 | 7.13 | 9.28 | 1244.19 | 3.74 | <0.005 | 12.42 |
| Surgery Performed (Y/N) | -5.43 | 0 | 2 | -9.35 | -1.5 | 0 | 0.22 | -2.71 | 0.01 | 7.22 |

| Concordance | 0.83 |
| --- | --- |
| Partial AIC | 237.9 |
| Log-likelihood Ratio Test | 75.45 on 7 df |
| -log2(p) of II-Ratio Test | 30.94 |
